# Relationship Between Wood Color Parameters Measured by the CIELab System and Extractive and Phenol Content in *Acacia mangium* and *Vochysia guatemalensis *from Fast-Growth Plantations

**DOI:** 10.3390/molecules17043639

**Published:** 2012-03-26

**Authors:** Róger Moya, Roy Soto Fallas, Pablo Jiménez Bonilla, Carolina Tenorio

**Affiliations:** 1 Instituto Tecnológico de Costa Rica, Escuela de Ingeniería Forestal, P.O. Box 159-7050 Cartago, Costa Rica; 2 Escuela de Química, Facultad de Ciencias Exactas y Naturales, Universidad Nacional de Costa Rica, Apartado 86-3000 Heredia, Costa Rica; Email: roysoto@costarricense.cr (R.S.F.); pabloijb@hotmail.com (P.J.B.); Tel.: +506-277-3579; Fax: +506-277-3349

**Keywords:** extractives, CIELab, phenols, tropical wood, wood color

## Abstract

**:** The heterogeneity of color distribution between sapwood and heartwood limits the market for wood from fast-growth plantations of tropical species. Wood color is associated with wood extractives contents. This study presents the relationship between wood color parameters measured by the CIELab color system and total amount of extractives and phenolic-type extractives in ethanol-toluene and hot water extracts of wood from two fast-growth plantation species. The results demonstrated that the difference in sapwood and hardwood color in *Vochysia guatemalensis* and *Acacia mangium* is caused by lower concentrations of extractives in sapwood of both species. Additionally, variations in total extractive and phenolic content have different effects on the color parameters (L*, a* and b*) of both species studied. In *Vochysia guatemalensis* wood, parameter L* decreases as total extractive and phenolic content increases; however, parameter a* increases as the content of extractives and phenols increases. In *Acacia mangium*, the amount of phenols showed no relationship with the color parameters. The ethanol-toluene total extractive content, however, shows a relationship with several color parameters. An increase in the content of total extractives in water and ethanol-toluene increases parameter a*, but decreases parameter L*.

## 1. Introduction

Wood color, together with physical and mechanical properties, is an important quality parameter because color is associated with decay resistance, commonly known as natural durability [1]. For example, it has been determined that in tropical species such as *Tectona grandis*, color measured using the CIELab system is related to resistance to degradation [2]. On the other hand, wood color plays a predominant role in the commercialization process [3,4], particularly when used for flooring or to make veneers or furniture [5]. Thus, wood color differences between sapwood and heartwood have limited commercialization of some tropical species due to irregular color.

The number of color determination techniques has increased over the last years [6] and these seek to create a series of quantitative parameters [7] that are later correlated with other wood properties [8]. The color determination has also concentrated on temperate species [2]. It has been demonstrated that wood color is dependent on species [4], tree genetic resources [9], silvicultural treatments [2,10], drying schedule applied [11] and wood preservation or thermal treatments [12]. However, all these wood color variations in tropical species result from variations in the amount and type of extractives present [13]. 

There is a poor understanding of the influence of extractives in tropical species. Extractives vary between and within trees and they are related to soil properties, tree age and environmental conditions where trees grow [2,15]. On the other hand, several studies suggest that the largest variations in wood color are associated to extractives content [2,11,14,15]. It has also been said that a large variety of extractives can be found in tropical species [16] and that the dark color of many of these species is the result of a high content of phenolic components [17,18,19]. Explanations provided are based on extractive content in some temperate species. For example, Gierlinger *et al.* [15] found that in several larch species the redness color (a*) and luminosity (L*) parameters correlate highly with the extractive content of wood, while the yellow color parameter correlates with the photochemistry of cell wall chemical components (cellulose, hemicellulose and lignin). Gierlinger *et al.* [15] mentioned that the correlation between wood color and extractives content are important to high heritability of extractives content, suggesting that chemical composition could be altered through tree breeding. Therefore color measurements on wood powder were a good indicator to phenolics and extractives content and may be useful in breeding for higher phenolic content and we can increase decay resistance of wood. But despite these claims, the relationship between wood color parameters measured and extractive content in tropical species has been limited to a few species, among these *Tectona grandis* [20].

In view of this, the objective of this study was to establish the relationship between wood color parameters of sapwood and heartwood and total amount of extractives and phenols in ethanol-toluene and hot water extracts of *Vochysia gatemalisis* and *Acacia mangium *wood from fast-growth plantations. These species are of great interest for commercial reforestation in some tropical regions [21,22], but their market is limited by the irregularity and color difference between sapwood and heartwood. 

## 2. Results

### 2.1. Wood Color

Average color parameter values, coefficient of variation (CV) and the minimum and maximum color parameter values measured according to the CIELab system in *V.* *guatemalensis* and *Acacia mangium* sapwood and heartwood are shown in Table 1. All color parameters were positive, with the exception of the a* parameter in *Acacia mangium *sapwood (Table 1). The results show that the wood color of these species is a combination of lightness, redness and yellowness components, with the exception of *A. mangium* sapwood, which has a dominance of greenness. Parameters L* and a* were statistically different in heartwood and sapwood of both species. The ANOVA analysis revealed that sapwood L* was higher than heartwood L* in both species and sapwood a* was lower. Sapwood and heartwood b*, however, showed no statistical difference between both species (Table 1). Parameter a* variations were the highest in sapwood and heartwood of both species, with a CV over 27%. exhibited moderate CV, ranging from 3.53% to 14.23% (Table 1).

**Table 1:** Color parameters of *Acacia mangium *and *Vochysia guatemalensis *using the CIELab System.

Wood color parameters	*Acacia mangium*		*Vochysia guatemalensis*	
	Sapwood	Heartwood	Sapwood	Heartwood
L*	84.1 ^A^ (1.86)	56.62 ^B^ (6.88)	80.56 ^A^ (1.61)	73.88 ^B^ (3.64)
	[83.13–86.43]	[49.75–68.34]	[78.91–82.78]	[69.08–78.56]
a*	−0.46 ^A^ (47.61)	4.11 ^B^ (22.87)	2.33 ^A^ (38.63)	4.65 ^B^ (32.25)
	[−1.29–0.04]	[2.01–5.63]	[1.11–3.79]	[2.70–7.45]
b*	22.38 ^A^ (3.53)	23.05 ^A^ (8.80)	15.77 ^A^ (9.00)	17.78 ^A^ (14.23)
	[21.75; 23.54]	[18.55–28.07]	[12.99–18.43]	[12.96–21.52]

Note: Minimum and maximum values are shown in square brackets and CV in parentheses. Average values identified with the letters A and B are statistically different at α = 99%.

### 2.2. Extractive Yields

EY obtained during the different extraction phases (first in an ET solution and then in HW) and total EY are summarized in Table 2. The highest EY values were obtained during HW extraction for both wood types in both species, ranging from 9.18 to 14.41%. *A. mangium *total EY was 11.36% in sapwood and 20.70% in heartwood, while in *V. guatemalensis* these values were 17.13% and 17.92%, respectively. *A. mangium* sapwood EY (in ET, HW and total EY) was lower than heartwood EY. In *V. guatemalensis*, however, no differences were found between sapwood and heartwood. In ET, variations in EY were highest in sapwood (CV = 36%) and heartwood (CV = 23%), while CV for the other wood conditions ranged from 10 to 18% (Table 2). 

### 2.3. Phenol Content

Extraction of *A. mangium* and *V. guatemalensis* sapwood and hardwood extractives first in HW and then using an ET solution revealed that the HW extract had the highest PC (first extraction phase). PC in HW and ET extracts and total PC were higher in *A. mangium* heartwood than in *A. mangium* sapwood; however, there was no statistically significant difference between these two wood types in *V. guatemalensis*. On the other hand, PC variations were higher than color parameters and extractive content (Table 1 and Table 2). CV values ranged from 24 to 50%. The coefficient of variation of PC ranged from 24 to 50% and these values were higher than values obtained for extractive content and color parameters (Table 1 and Table 2). The CV of PC in *A. mangium* and *V. guatemalensis* exhibited no defined behavior according to wood type (Table 2). 

**Table 2:** Extractives in ET and HW *and* phenol content in ET and HW extracts for *Acacia mangium *and *Vochysia guatemalensis*.

Type	*Acacia mangium*		*Vochysia guatemalensis*	
	Sapwood	Heartwood	Sapwood	Heartwood
Extractives in ET (%)	2.18 ^A^ (36)	6.29 ^B^ (23)	3.44 ^A^ (16)	3.93 ^A^ (13)
	[1.31–2.98]	[4.24–9.11]	[2.93–4.62]	[2.85–4.79]
Extractives HW (%)	9.18 ^A^ (16)	14.41 ^B^ (13)	13.69 ^A^ (10)	13.99 ^A^ (12)
	[7.69–11.20]	[11.69–18.06]	[11.03–15.26]	[11.47–17.56]
Total extractives yield	11.36 ^A^ (18)	20.70 ^B^ (11)	17.13 ^A^ (10)	17.92 ^A^ (10)
	[9.00–13.89]	[17.29–25.27]	[14.05–19.32]	[14.84–22.16]
Phenols in HW	966 ^A^ (39)	6261 ^B^ (28)	1922 ^A^ (35)	2289 ^B^ (32)
	[560–1472]	[3256–10243]	[689–2896]	[1089–3658]
Phenols in ethanol-toluene	324 ^A^ (50)	3239 ^B^ (44)	441 ^A^ (30)	423 ^A^ (50)
	[167–552]	[754–6166]	[221–657]	[90–795]
Total phenol content	1290 ^A^ (40)	9500 ^B^ (24)	2362 ^A^ (31)	2712 ^A^ (29)
	[832–2025]	[4009–13902]	[980–3320]	[1331–4132]

Note: Minimum and maximum values are shown in square brackets and CV in parentheses. Average values identified with the letters A and B are statistically different at α = 99%.

### 2.4. Relationship between Wood Color Parameters and Extractives and Phenol Content

**Figure 1:**
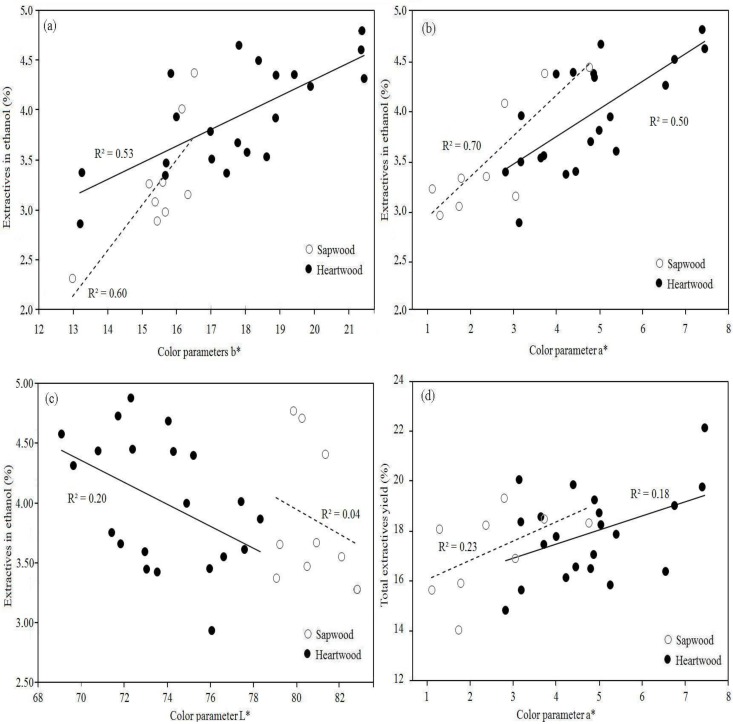
Relationship between *extractives* in ethanol and the b* and a* color parameters in sapwood and heartwood of *Vochysia guatemalensis*.

The coefficient of correlation, considering sapwood and heartwood together, between color parameters and EY in HW, ET and total EY are detailed in Table 3. No relationship was found between HW extractives and PC with color parameters in *V. guatemalensis* for all samples (Table 3). The same result was found when sapwood and heartwood were considered as separated samples. However, ET extractives exhibited a relationship with all color parameters in this species for all samples and the coefficients of determination ranged from −0.53 to 0.76 (Table 3). Regression analysis for sapwood and heartwood showed too that ET extractives were positively correlated with a* and b* color parameters. The values of parameters a* and b* increased significantly with the increment in extractives in the ET extracts in both type of wood (Figure 1a and 1b). Parameter L* in sapwood and heartwood, on the other hand, was too affected for extractives in ET solvent. This parameter decreased with the increase in extractives in this solvent (Figure 1c). The total EY only was positively correlated with parameter a* in sapwood and heartwood of *V. guatemalensis *(Figure 1d). 

**Table 3:** Pearson’s correlation *between* wood color parameters and extractives content in *V. guatemalensis* (upper diagonal) and *A. mangium* (lower diagonal), considering sapwood and heartwood samples together.

Parameters	L*	a*	b*	Extractives in hot water	Extractives in ethanol-toluene	Total extractives yield	Phenols in hot water	Phenols in ethanol-toluene	Total phenol content
L*	1	−0.71 **	−0.51 **	0.05 ^NS^	−0.53 **	−0.13 ^NS^	−0.15 ^NS^	0.15 ^NS^	−0.11 ^NS^
a*	−0.92 **	1	0.64 **	0.25 ^NS^	0.76 **	0.48 *	0.02 ^NS^	0.14 ^NS^	0.06 ^NS^
b*	0.12 ^NS^	−0.05 ^NS^	1	0.04 ^NS^	0.70 **	0.26 ^NS^	−0.04 ^NS^	0.01 ^NS^	−0.03 ^NS^
Extractives in hot water	−0.68 **	0.71 **	0.18 ^NS^	1	0.68 **	0.95 **	0.01 ^NS^	0.48 **	0.13 ^NS^
Extractives in ethanol-toluene	−0.82 **	0.81 **	−0.14 ^NS^	0.68 **	1	0.47 **	−0.14 ^NS^	0.12 ^NS^	−0.10 ^NS^
Total extractives yield	−0.80 **	0.82 **	0.05 ^NS^	0.94 **	0.68 **	1	−0.0 ^NS^	0.47 **	0.08 ^NS^
Phenols in hot water	−0.69 **	0.60 **	−0.24 ^NS^	0.37 *	0.69 **	0.55 **	1	0.17 ^NS^	0.97 **
Phenols in ethanol-toluene	−0.82 **	0.69 **	0.05 ^NS^	0.57 **	0.53 **	0.61 **	0.46 **	1	0.40 *
Total phenol content	−0.72 **	0.62 **	−0.28 ^NS^	0.39 **	0.70 **	0.57 **	0.91 **	0.51 **	1

Legend: ** statistically significant at the 99% confidence level; * statistically significant at the 95% confidence level.

**Figure 2:**
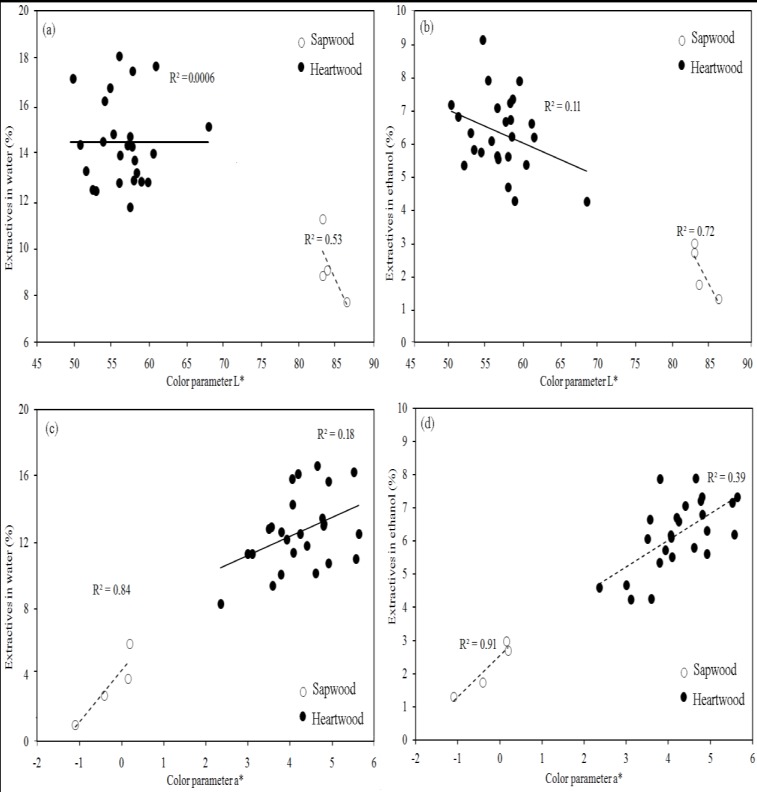
Relationship between *extractives* in water and ethanol and the L* and a* color parameters in *Acacia mangium*.

It was found that parameters L* and a* in *A. mangium* were statistically related to extractives in HW and ET extracts and total EY and the coefficient of correlation ranged from −0.68 to 0.82 when sapwood and heartwood are considered together (Table 3). However, when sapwood and heartwood were analyzed separately, heartwood was not related with L* (Figure 2a), while L* of sapwood exhibited a negative relationship with extractives in HW extract (Figure 2a) and ET extract was again negatively correlated with L* parameters of sapwood and heartwood (Figure 2b). Parameter a* relationships, on the other hand, are contradictory to those obtained for parameter L*. Parameter a* was positively related to extractives in HW and ET extracts (Figure 2c and Figure 2d, respectively) in sapwood and heartwood. 

**Figure 3:**
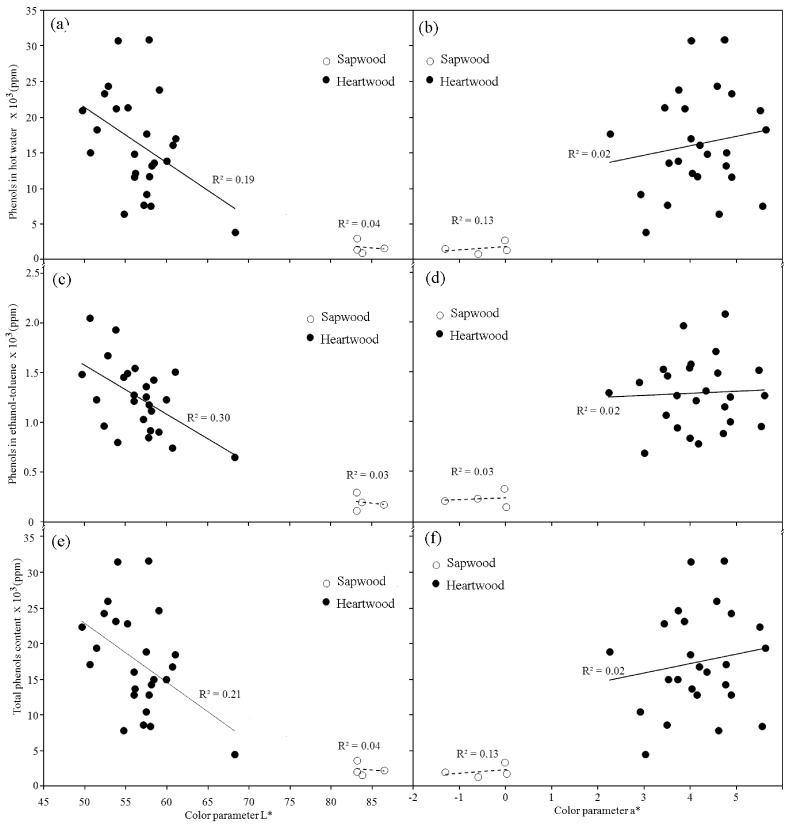
Relationship between phenol content using tannic acid and color parameters in *Acacia mangium* wood.

PC in HW and ET extracts and total PC was correlated with L* and a* in *A. mangium* when all samples were analyzed together. The coefficients of correlation ranged from −0.82 to 0.82 (Table 3). However, not correlation was found between a* parameters and PC (in water, in ethanol-toluene solution or total of phenol) when sapwood and heartwood were analyzed separately (Figure 3b,d,f). A negative relationship between those extractives and parameter L* were found heartwood, but any relation was found wood (Figure 3a,c,e).

## 3. Discussion

The color of *A. mangium *wood varied and there was a difference in heartwood and sapwood color, as expected (Table 1). This color difference is due to the synthesis and accumulation of extractives during heartwood formation [5]. Heartwood color is due in part to oxidation and polymerization reactions during the aging process of the tree [15,17,23,24]. The high values of EY and PC in HW and ET in *A. mangium *(Table 2) corroborate these studies, which establish that wood color is related to extractive amount and type. In the case of *V. guatemalensis *wood, however, the latter does not apply. Despite a statistical difference in heartwood and sapwood L* and a* parameters, the amount of heartwood and sapwood extractives and phenols did not vary in all cases (Table 2). Nonetheless, a statistical difference in sapwood and heartwood PC was found in HW extract (Table 2), which is likely responsible for the slight difference in color between heartwood and sapwood of this plantation species. This slight difference between heartwood and sapwood EY and PC in *V. guatemalensis* might also be influenced by tree age. Trees sampled in this study were between 8 and 10 years old when harvested and due to their young age the difference in heartwood and sapwood color is only slight. In older trees, however, this slight difference in color may become more accentuated since extractive content increases with tree age [24,25].

Wood color measured by the CIELab system revealed that high levels of lightness (L*), moderate levels of yellowness (b*) and low levels of redness (a*) are characteristic of *A. mangium* and *V. guatemalensis* sapwood. In heartwood, however, lightness (L*) decreases and redness (a*) increases slightly. Several tones of greenness were found in *A. mangium* sapwood and the average value of parameter a* was −0.46. Another important difference is that *A. mangium* heartwood is darker than *V. guatemalensis* heartwood. Low L* values and high a* values account for darker *A. mangium* heartwood (Table 1). Sapwood color in *V. guatemalensis*, on the other hand, is lighter than in *A. mangium.* Despite the fact that *A. mangium* exhibited the highest lightness (L*) and yellowness (b*) values, the presence of greenness (−a*) results in darker sapwood.

On the other hand, color parameters and extractive and phenolic content (in HW and ET extracts) for both species varied widely, especially parameters a* and b* and EY (Table 2). One of the possible causes for this variation is the varied origin of the trees, which came from 30 different plantations located in two different regions of Costa Rica. Wood color can also vary due environmental differences or silvicultural treatments [2,10,26,27]. Despite variations in all wood color parameters, several studies suggest that these variation are caused by variations in parameter a* followed by variations in parameter b* [2,11,14,15]. As with wood color, EY varies greatly between sites [27,28] and therefore, the high variability found in *A. mangium* and *V. guatemalensis *EY could be influenced by the tree’s place of origin.

*A. mangium* heartwood PC was higher than in *V. guatemalensis *heartwood (Table 3) and heartwood color was dark in *A. mangium* and light in *V. guatemalensis*. The color difference between sapwood and heartwood is once again due to the presence of PC [19]. Tropical species contain a large variety of extractives [16] and may affect wood color differently. For example, bioactive components called tectoquinones produce black streaks along the annual rings in *Tectona grandis* [20]. Thus, the color difference between sapwood and heartwood is probably the result of non-phenolic extractives in *V. guatemalensis* wood that were not considered in this study. 

Color parameters of sapwood or heartwood were less affected in *V. guatemalensis* in relation to *A. mangium*. However, several other factors might also be affecting heartwood color in *V. guatemalensis* wood since it is slightly darker than sapwood. Extractives in HW and ET or total EY (Table 1) were similar in both types of wood (Table 1) and there was no relationship between color parameters and PC (Table 3). On the hands, the extractives effects were similar in sapwood and heartwood of *V. guatemalesis*, but there were different in *Acacia mangium*. For example, extractives content in ET increased the b* and a* values in same way both heartwood and sapwood of *V. guatemalensis* (Figure 1a and 1b). But these relationships were different in sapwood and heartwood of *A. mangium*, extractives en HW did not produced effects in heartwood, but this extractives were negatively correlated with L* color parameter (Figure 2a).

A darker heartwood color is associated with a high PC [17,18,19]. This is true in the case of *A. mangium *heartwood. The lightness (L*) increased with decreasing of PC in HW and ET (Figure 3a,c,d) and light increasing was found in redness (a*) color with increasing of PC (Figure 3b,d,f). These relationship means that darker color will be present in heartwood when higher PC is present. However, although phenol extractives are present in sapwood (Table 2), any relationships between PC and wood color parameters was found in sapwood of *A. mangium *(Figure 3a–f). Probably the lack of relationship is influenced by lower PC in sapwood in relation to PC of heartwood (Table 2).

The correlation found between PC and parameters L* in *A. mangium* heartwood (Figure 3a,c,e) coincides with other studies [15,17,18]. For example, two species of Juglans (*J. nigra* and J. hybrid *J. nigra* × *J. regia*) exhibited a positive correlation between parameter b* and PC [17]. The same behavior was found in European oak wood [14], different larch species [15] and in Douglas-fir [18]. 

*Acacia mangium*, as well as other species of the genus *Acacia*, is characterized by a large amount of substances in the wood structures [29,30] that form during heartwood formation and collect inside the vessels [31]. These substances are numerous and include amines and alkaloids, cyanogenic glycosides, cyclitols, fatty acids and seed oils, fluoroacetates, gums, non-protein amino acids, terpenes (essential oils, diterpenes, phytosterol and triterpene genins and saponins), hydrolysable tannins, flavonoids and condensed tannins. Polysaccharides (gums) and complex phenolic substances (condensed tannins) are the most evident and best known [32]. It is likely that all these substances influence wood color in one way or another.

## 4. Experimental

*Wood samples*: 30 *Vochysia guatemalensis* samples (from 8−10 year old trees) and 30 *Acacia mangium* samples (from 7−10 year old trees) were obtained from 30 different trees from each species. These trees came from 30 different plantations located in two different regions of Costa Rica. Samples were extracted from kiln−dried boards chosen at random and used in several different studies conducted by the Instituto Tecnológico de Costa Rica. Previous publications detail site, management conditions and age of trees sampled [11,21,22]. Samples measured 2 × 2 × 2 cm and were taken from the center of the boards (half of the length) and included sapwood and heartwood. In the *A. mangium *boards, demarcation between sapwood and heartwood is well defined and therefore, sample extraction was easy; however, demarcation between these two types of wood in juvenile trees was not apparent in the *V. guatemalensis* boards, which made sample extraction slightly more difficult. Wood color of heartwood is slighter darker than sapwood and heartwood is produced about 4-year-old in this tropical species. Samples were of tangential, radial and longitudinal orientation and these were stored at a temperature of 20 °C and a relative humidity of 65% until an equilibrium moisture content of 12% was reached.

*Wood color determination*: It was measured on two tangential faces of sample board. A HunterLab MiniScan® XE Plus spectrophotometer was used and color parameters were determined using the CIELab system. This system estimates wood color using the three spatial coordinates L*, a* and b* [33]. L* represents lightness y measures the position on the black-white axis (L = 0 for black and L = 100 for white), a* represents the chroma value and defines the position on the red-green axis (+100 values for red shades, −100 values for green shades) and b* represents the chroma value and defines the position on the yellow–blue axis (+100 values for yellow shades, −100 values for blue shades). Color measurement conditions were: wave length range between 400 and 700 nm, with a 13 mm aperture at the point of measurement. The specular component (SCI mode) was included in order to observe reflection at a 10° angle, which is normal for the specimen surface (D65/10), as well as a 2° field of vision (standard observer, CIE 1931) and standard D65 illumination, which corresponds to daylight at 5,500K.

*Successive extraction with ethanol and hot water*: After color was measured, wood samples (10 g) were milled to a particle size of less than 0.6 mm and then screened through a 40- and a 60-mesh. Particle size collected was between 40 and 60 mesh. Samples screened were divided into two parts: 1 g to determine moisture content and two 2 g samples for successive extraction with an ethanol-toluene (ET) solution and then hot water (HW). Extractives were extracted and quantified according to ASTM Standard D1105−96 [34]. The ET solution was prepared with 1 liter of ethanol and 0.427 liters of toluene and 50 mL of this solution were added to a 2 g screened sample. The extraction procedure lasted six hours and was conducted using the Soxtec^TM^ 2043 Extraction System manufactured by Foss Tecator. The extract was then separated from the sample and stored, while the wood sample was refluxed for one hour in 250 mL of HW. A No. 3 sintered glass filter was used to filter the ET and HW extracts and the filtered material was kept in opaque glasses. Extractive yields (EY) and total EY were calculated for both the ET and HW extractives. Moisture content was determined according to ASTM Standard 2395−02 [35] and was used to correct extractive value in accordance with ASTM Standard D1105−96 [34].

*Phenol content*: Folin−Ciocalteu reagent was used to measure phenolic content [36]. This standard reagent was prepared with sodium tungstate, sodium molybdate, lithium sulfate, bromine, phosphoric acid and chloric acid reagents. Phenol content (PC) was determined in the ET and HW extracts, and measured according to the following procedure: (i) Both HW extracts (250 mL each) were combined into a single sample (500 mL) and both ET extracts were also combined as a single sample (100 mL); (ii) The HW extract was kept in the 500 mL solution. However, the ET extract was separated from the solution using a rotary evaporator and the precipitate was dissolved in 250 mL of water; (iii) Then, a 0.1 mL aliquot was extracted from this solution and dissolved in 7.9 mL of water and 0.5 mL of Folin-Ciocalteu reagent; (iv) This solution remained still for 8 minutes and then 1.5 mL of 20% sodium carbonate solution (Na_2_CO_3_) were added. A UV-VIS spectrophotometer (T18 manufactured by PG Instruments) was used to determine the amount of phenols in this sample. PC estimations in the wood samples were calculated by multiplying extractive yield by the phenolic content values obtained spectrophotometrically. Finally, PC was calculated in relation to initial sawdust weight and expressed as a percentage.

*Data analysis*: Statistical analysis was conducted using SAS 8.1 software (SAS Institute, Inc., Cary, NC, USA). EY was reported during extraction with ET and then HW. Total EY was calculated as the sum of extractive values in ET and HW. The color parameters (L*, a* and b*) of both tangential faces of the sample were averaged out and color was established for sapwood and heartwood in both species. EY from both screened samples was once again averaged out and calculated for the sapwood and heartwood samples. The one-way ANOVA (analysis of variance) procedure was used to establish the difference between sapwood and heartwood in both species. Additionally, Tukey’s test was applied to establish differences between sapwood and heartwood total EY and EY means. Phenolic content (PC) was calculated separately in the ET solution and in HW, as well as total PC, which is equivalent to the sum of PC in ET and HW. Pearson’s correlation coefficients were used to determine the relationship between wood color parameters and PC and total PC, as well as EY and total EY. Correlation coefficients were calculated for together sapwood and heartwood samples, since the sapwood samples were few to establish a correlation with wood color parameters.

## 5. Conclusions

The data discussed in this paper suggests that the difference in color between *Vochysia guatemalensis* and *Acacia mangium* sapwood and heartwood is mainly the result of a lower concentration of extractives in sapwood of both species. Also, the variation in total EY in ET and in HW has a different effect on the color parameters (L*, a* and b*) in wood with little differentiation between sapwood and heartwood such as *Vochysia guatemalensis*, as opposed to wood where sapwood and heartwood are well differentiated such as *Acacia mangium*. 

In *Acacia mangium *wood, parameter L* decreases when total EY and PC increase, and parameter a* increases when EY and PC increase. In *Vochysia guatemalensis*, on the other hand, PC exhibited no relationship with color parameters, but total EY in ET did exhibit a relationship with parameters a* and L*. An increase in total EY in HW and ET increases the value of parameter a* and decreases parameter L*.
